# Complete Genome Sequence of *Kyrpidia* sp. Strain EA-1, a Thermophilic Knallgas Bacterium, Isolated from the Azores

**DOI:** 10.1128/genomeA.01505-17

**Published:** 2018-01-18

**Authors:** Johannes Eberhard Reiner, Christian Jonas Lapp, Boyke Bunk, Cathrin Spröer, Jörg Overmann, Johannes Gescher

**Affiliations:** a Department of Applied Biology, Institute for Applied Biosciences, Karlsruhe Institute of Technology (KIT), Karlsruhe, Germany; b Deutsche Sammlung für Mikroorganismen und Zellkulturen GmbH (DSMZ), Braunschweig, Germany

## Abstract

*Kyrpidia* sp. strain EA-1 is a thermophilic hydrogen-oxidizing bacterium isolated from hydrothermal systems at São Miguel Island, Portugal. Here, we present the complete genome sequence of the strain assembled to a single circular chromosome. The genome spans 3,352,175 bp, with a GC content of 58.7%.

## GENOME ANNOUNCEMENT

So far, the only known member of the genus *Kyrpidia*, *K. tusciae*, is a Gram-positive rod-shaped bacterium capable of chemolithoautotrophic and chemoorganoheterotrophic growth. As an autotrophic thermophile, *Kyrpidia tusciae* is able to assimilate CO_2_ in extreme environments (1, 2). A new *Kyrpidia* strain was isolated at 60°C from hydrothermal sediment samples collected at São Miguel Island, Portugal. The genome of this aerobic hydrogen-oxidizing strain, *Kyrpidia* sp. EA-1, was sequenced in order to gain a deeper insight into carbon dioxide fixation at high temperatures.

Genomic DNA was isolated using the Wizard genomic DNA purification kit (Promega, Mannheim, Germany), according to the manufacturer’s instructions. The concentration of double-stranded DNA (dsDNA) was measured with a Qubit dsDNA BR assay kit (Thermo Scientific, Darmstadt, Germany). Whole-genome sequencing was conducted using a dual sequencing approach; PacBio single-molecule real-time (SMRT) sequencing technology was combined with a Nextera XT library preparation kit and an Illumina MiSeq PE250 instrument (Illumina, Eindhoven, The Netherlands). A SMRTbell template library was prepared according to the instructions from Pacific Biosciences (Menlo Park, CA, USA) (see http://www.pacb.com/wp-content/uploads/2015/09/Procedure-Checklist-10-kb-Template-Preparation-and-Sequencing.pdf). Briefly, for the preparation of 15-kb libraries, 3.5 µg of genomic DNA was end repaired and ligated overnight to hairpin adapters, applying components from the DNA/polymerase binding kit P6 from Pacific Biosciences. BluePippin size selection to greater than 4 kb was performed according to the manufacturer’s instructions (Sage Science, Beverly, MA, USA). The conditions for annealing of the sequencing primers and binding of polymerase to the purified SMRTbell template were assessed with the calculator in RS Remote (Pacific Biosciences). SMRT sequencing was carried out on the PacBio RSII (Pacific Biosciences), taking one 240-min movie for one SMRT cell using P6 chemistry. Sequencing resulted in 45,709 postfiltered reads, with a mean read length of 10,937 bp. SMRT cell data were assembled using the RS_HGAP_Assembly.3 protocol included in SMRT Portal version 2.3.0, using default parameters. The assembly revealed a circular chromosome. The validity of the assembly was checked using the RS_Bridgemapper.1 protocol. The chromosome was circularized, and particularly, artificial redundancies at the ends of the contigs were removed and adjusted to *dnaA* as the first gene. Error correction was performed by a mapping of 1 million paired-end Illumina reads of 2 × 250 bp onto the finished genome using BWA (3), with subsequent variant and consensus calling using VarScan (4). A consensus concordance of QV60 could be confirmed for the genome. Finally, an annotation was carried out using the NCBI’s Prokaryotic Genome Annotation Pipeline (5).

The complete genome of *Kyrpidia* sp. strain EA-1 consists of a single circular chromosome with 3,352,175 bp and a GC content of 58.7%. The PGAP annotation tool revealed 3,087 protein coding sequences, 58 tRNA genes, and 15 rRNA genes.

### Accession number(s).

The complete genome sequence of *Kyrpidia* sp. strain EA-1 was deposited at NCBI GenBank under accession number CP024955.
